# Risk stratification of pediatric chest pain: red flags, diagnostic yield, and an evidence-informed clinical framework

**DOI:** 10.3389/fped.2026.1988473

**Published:** 2026-10-09

**Authors:** Lingling Meng, Wenjing Zhu, Jianli Lv

**Affiliations:** 1 Department of Emergency, Jinan Children's Hospital, Jinan, Shandong, China; 2 Department of Pulmonary and Critical Care Medicine, Shandong Provincial Hospital Affiliated to Shandong First Medical University, Jinan, Shandong, China; 3 Department of Pediatric Cardiology, Shandong Provincial Hospital Affiliated to Shandong First Medical University, Jinan, Shandong, China

**Keywords:** adolescent, chest pain, child, point-of-care systems, risk assessment

## Abstract

Chest pain in children and adolescents is usually noncardiac, but uncommon cardiac and other serious conditions require timely recognition. Existing reviews describe etiologies and diagnostic investigations; translating this evidence into decisions across clinical settings remains challenging. This narrative review integrates pediatric chest-pain cohorts, diagnostic studies, pathway evaluations, and professional guidance to clarify how clinical context should influence testing and disposition. A reproducible, focused literature update was performed on 27 September 2026 using PubMed records accessed through Europe PMC, with additional verification of references and institutional guidance. Evidence was interpreted according to study design, population selection, and applicability rather than pooled into a new effect estimate. Observational studies support history- and examination-directed evaluation, but heterogeneous outcome definitions, selective testing, and limited follow-up constrain conclusions about safety. We propose an assessment framework separating physiological instability, stable presentations with cardiac warning features, stable presentations suggesting a noncardiac disorder, and provisionally low-risk presentations. Low risk is a conditional clinical assessment requiring an adequate history, reassuring vital signs and examination, and no concerning disease-specific features; it is not a quantified probability or a discharge rule. Conservative outpatient management additionally requires a workable reassessment plan. Troponin testing should address a plausible concern for myocardial injury, and its interpretation depends on pretest probability, assay characteristics, and symptom timing. Point-of-care ultrasound is an optional, expertise-dependent adjunct with limited direct evidence in unselected pediatric chest pain. Clinical setting, access to investigations and specialists, and follow-up reliability affect the appropriate next step. The framework organizes existing evidence and identifies implementation gaps; it has not been derived or validated as a clinical decision rule and requires prospective evaluation before protocol-level adoption.

## 1 Introduction

Chest pain is a frequent reason for pediatric emergency, primary care, and cardiology assessment. Although most presentations are benign, families may associate chest pain with sudden cardiac death. Clinicians must recognize uncommon serious disease while avoiding investigations that are unlikely to answer a relevant diagnostic question. Recent narrative reviews have described the etiologic spectrum and the performance and limitations of available tests (1, 2). These reviews support selective evaluation, but the practical meaning of low risk and the transferability of recommendations between settings need further clarification.

The incremental contribution of this review is a context-sensitive synthesis of those decisions. We distinguish direct pediatric chest-pain evidence from evidence in selected populations, professional guidance, and our own proposed implementation steps. We make the conditions underlying a provisional low-risk assessment explicit, place troponin within a pretest-probability framework, and connect disposition to local diagnostic capability and access to reassessment. These are refinements in interpretation and application, not newly discovered predictors or proof that the proposed framework improves outcomes. Existing etiologic classifications and established pathways remain the foundation of the review.

The framework primarily concerns nontraumatic chest pain in children and adolescents who can describe their symptoms. Major trauma, infants with nonspecific distress, and patients with known complex cardiac disease require their own assessment pathways; the general principles of physiological stabilization still apply. Studies enrolling young adults alongside children are identified where this affects applicability. Neither a fixed age cutoff nor an adult acute coronary syndrome algorithm can substitute for assessment of the individual child (3, 4).

## 2 Search strategy and evidence interpretation

### 2.1 Scope and reproducible literature update

This is a narrative review, not a systematic review or a diagnostic-rule derivation study. The earlier manuscript and its bibliography served as the starting reference set. For this revision, focused searches were executed on 27 September 2026 in Europe PMC, restricted to its PubMed source records (SRC:MED), English-language publications, and first publication dates from 1 January 2000 to 27 September 2026. Three complementary queries addressed pediatric chest pain, pediatric troponin and myocardial injury, and pediatric cardiac or lung point-of-care ultrasound (POCUS). The chest-pain query used the phrase in the title and pediatric population terms in titles or abstracts; the supplementary queries used title/abstract terms. PubMed source records accessed through Europe PMC are not equivalent to an independently executed search of every MEDLINE interface. No completed Embase, Web of Science, or Cochrane Library search is claimed in this revision.

Records were selected purposively for their relevance to etiology, clinically important outcomes, warning features, diagnostic yield, or pathway implementation. Priority was given to pediatric observational cohorts, diagnostic studies, and implementation evaluations; published reviews provided context, and professional statements and institutional guidance informed practice considerations. Adult-only studies and isolated case reports were not used to estimate pediatric prevalence or test performance. Mixed pediatric/young-adult cohorts were retained when informative, with their age range acknowledged. Bibliographic details were checked against indexed records, and available full texts were examined for key design limitations. The Royal Children's Hospital chest-pain guideline and American College of Radiology (ACR) guidance were also consulted (5, 6).

Selection and synthesis were not conducted as independent duplicate systematic-review screening. There was no registered protocol, formal risk-of-bias scoring, GRADE assessment, or new meta-analysis. Search retrieval counts therefore describe the update, not a PRISMA study-selection flow. The title-restricted chest-pain search, English-language limit, reliance on one bibliographic source, selective inclusion, and incomplete access to some full texts may have omitted relevant evidence. These limitations constrain both the review's comprehensiveness and the strength of its clinical suggestions.

### 2.2 How evidence informs the framework

We distinguish four sources of support. Direct evidence comprises pediatric chest-pain cohorts and pathway studies. Indirect or selected-population evidence includes troponin-tested cohorts, children with preexisting heart disease, and studies targeting a particular diagnosis. Professional guidance combines available evidence with expert interpretation. Finally, the arrangement of the proposed framework and its practical adaptations are an author synthesis, not findings from an implementation trial. These categories describe provenance and applicability, not formal certainty grades.

Greater weight is given to evidence that addresses the relevant population and clinical outcome. A large selected cohort may still be less informative for an unselected child than a smaller directly applicable study. Diagnostic yield, sensitivity, and a management benefit are separate outcomes: a low rate of abnormal tests does not establish that testing can safely be omitted, and a high negative predictive value in a low-prevalence population does not independently validate a rule. Conditional language is used for testing and follow-up suggestions. Immediate treatment of physiological compromise remains a general emergency-care principle rather than a conclusion newly established by this narrative review. Table 1 provides clinical orientation, and Table 2 maps pivotal evidence to the implications it can and cannot support.

**Table 1:** Clinical patterns and conditional assessment options for pediatric chest pain.

Clinical pattern	Features requiring greater concern	Conditional assessment and management	Supporting sources
Musculoskeletal or chest-wall pain	Significant trauma, swelling, persistent focal bony pain, systemic illness, or independent cardiac warning features	Reproducible pain supports a chest-wall explanation but is not a rule-out test. Symptom relief and review may suffice after a reassuring assessment; choose imaging for unresolved concern and local guidance.	(1, 6, 12, 14)
Precordial catch syndrome	Atypical duration, exertional syncope, ongoing respiratory symptoms, or abnormal examination	A typical brief focal episode in an otherwise well child may be managed with explanation and reassurance. Reassess an atypical or changing pattern; avoid categorical no-testing advice.	(1, 3, 5)
Idiopathic or nonspecific pain	Unexplained progression, impaired function, unreliable history, or new objective findings	Use a provisional working diagnosis after adequate assessment, rather than exhaustive testing. Agree on symptom review and return precautions.	(3, 8)
Anxiety-related or functional symptoms	Associated safety concerns, severe functional impairment, or physical findings not explained by the working diagnosis	Validate symptoms and assess psychosocial context. Coordinate primary care or mental health support when indicated; psychological symptoms do not exclude organic disease.	(15)
Asthma, pneumonia, or pleuritis	Hypoxemia, increased work of breathing, fever with systemic illness, or focal chest findings	Use pulse oximetry and condition-directed assessment. Radiography is guided by the diagnostic question; lung POCUS is an optional adjunct where expertise and local practice support it. Disposition follows severity.	(5, 6, 14)
Pneumothorax or pneumomediastinum	Sudden pleuritic pain, asymmetric breath sounds, subcutaneous emphysema, or physiological compromise	Urgent clinical assessment; treat suspected tension physiology without waiting for imaging. In stable patients, radiography is the usual initial imaging approach. Expert POCUS may supplement, not universally replace, the indicated assessment.	(6, 28)
Myocarditis or pericarditis	Persistent symptoms, systemic illness, disproportionate tachycardia, poor perfusion, ECG changes, arrhythmia, or heart-failure signs	ECG and troponin when myocardial injury is suspected; formal echocardiography and specialist input according to the presentation. Neither a normal initial troponin nor a negative POCUS scan independently excludes disease.	(3, 16, 18)
Possible arrhythmia	Sustained palpitations, syncope, abnormal pulse, known channelopathy, or concerning family history	ECG during symptoms where feasible; monitoring or ambulatory recording when clinically indicated. Treat instability promptly and select disposition according to the rhythm, symptoms, and available follow-up.	(3, 26)
Cardiomyopathy or coronary anomaly	Exertional pain or syncope, reduced exercise tolerance, pathological murmur, relevant family history, or known cardiac disease	ECG and cardiology-directed formal imaging. A normal ECG or limited POCUS does not exclude a coronary anomaly. Consider avoidance of strenuous activity pending appropriate assessment.	(3, 4)
Possible pulmonary embolism	Compatible symptoms with thrombotic risk, unexplained hypoxemia, syncope, or hemodynamic compromise	Use a pediatric specialist-guided diagnostic approach. Adult rule-out criteria should not be assumed to apply. Imaging and treatment depend on the specific concern and severity.	(6, 14)
Gastrointestinal, esophageal, or systemic disease	Dysphagia, drooling, possible battery ingestion, severe post-vomiting pain, or sickle cell disease with fever or respiratory symptoms	Uncomplicated reflux may be assessed clinically; obstruction or perforation requires urgent specialty care. Evaluate sickle cell presentations for acute chest syndrome using the relevant disease pathway.	(5, 6, 14)

This is a clinical orientation table, not a validated diagnostic or treatment rule. Sources include observational evidence and professional guidance; the conditional actions are a narrative synthesis. Apply the full assessment in Section 5.2 before considering a presentation provisionally low risk. ECG, electrocardiography; POCUS, point-of-care ultrasound.

**Table 2:** Selected evidence underlying the assessment framework and its limits.

Evidence and population	Finding relevant to this review	Main applicability or design limitation	Implication for the framework
Drossner et al. (7); direct observational evidence; two pediatric EDs; 4,288 visits after exclusions	Cardiac etiology in 24 visits (0.6%).	Retrospective discharge diagnoses; children with known cardiovascular disease excluded.	Low background frequency supports selective assessment, not a zero-risk assumption.
Okyere et al. (11); direct prehospital evidence; 4,277 ambulance attendances	Abnormal initial physiology was associated with serious short-term outcomes.	Linked subsets were used for outcomes; ambulance attendances are selected; composite outcome is not cardiac disease alone.	Prioritize physiological assessment. Do not derive a nontransport rule from these associations.
Friedman et al. (4); retrospective cardiology cohort; 406 referrals	Retrospective algorithm application projected reductions in selected tests.	Projected effects in a referred population; all patients underwent ECG.	Inform testing questions; do not present projected savings as observed benefit of the new framework.
Verghese et al. (30); SCAMP implementation; 364 patients versus 406 historical controls	Exercise testing and rhythm monitoring decreased; overall echo use did not change significantly.	Nonrandomized historical comparison in specialist care; secular and implementation effects possible.	Benefits are test- and setting-specific, not a universal percentage reduction.
Mohan et al. (32); ED before-and-after study; 1,687 eligible patients	Chest-radiograph ordering decreased; other testing and cardiology consultation did not materially change.	Single system, nonrandomized design, and incomplete within-system follow-up.	Supports local pathway evaluation, not proof of zero missed disease.
Primary care quality improvement (33); 421 presentations in four practices	Low-probability referrals fell from 17% to 5%; no life-threatening cardiac event was identified during reported follow-up.	Multicomponent intervention, limited setting and follow-up, few expected serious events.	Primary care implementation is feasible; transportability requires local evaluation.
Brown et al. (19) and Brancato et al. (18); selected pediatric troponin cohorts	Test interpretation depended on the clinical presentation and accompanying findings.	Clinician-selected testing and differing assays; yield is not sensitivity in an unselected population.	Request troponin for suspected myocardial injury rather than chest pain alone.
Genadry et al. (24); retrospective myocarditis case-control study; 93 cases and 202 controls	At least one of chest pain, fever, or specified ECG changes had 99% sensitivity and 14% specificity.	Selected tested population; case-control sampling; chest pain is itself a predictor.	Cannot identify a low-risk subgroup using that threshold in patients all presenting with chest pain.
Pediatric troponin reference and clinical cohorts (22, 23); indirect threshold evidence	Reference limits differ by assay; lower pediatric thresholds can increase injury classification.	A reference percentile or reclassification is not an outcome-validated treatment or discharge threshold.	Use assay-specific interpretation; do not adopt a universal cutoff from this review.
Lung POCUS pilot (28); 77 children selected for suspected pneumothorax	Thirteen pneumothoraces; encouraging accuracy in experienced hands.	Operator-dependent enrollment, small sample, enriched disease prevalence, and radiographic reference standard.	Optional focused adjunct; no support for universal POCUS screening or discharge clearance.
Hoffmann et al. (29); cardiac POCUS in 104 children with preexisting cardiac disease	Good agreement with expert POCUS review for effusion and systolic dysfunction.	Convenience sample with known disease; comparator was expert image review, not comprehensive exclusion of all pathology.	Do not transfer performance estimates to previously healthy, unselected chest-pain patients.
AHA, ASE, ACR, and institutional guidance (5, 6, 16, 27); professional recommendations	Provide disease-specific evaluation, imaging, training, and escalation principles.	Include expert interpretation; differing scope and incomplete primary evidence; do not validate this framework.	Apply relevant guidance locally and make any divergence or uncertainty explicit.

This is a selective evidence map, not an exhaustive systematic-review evidence table or formal GRADE assessment. Directness, design, and limitations should be considered together. Framework organization, discharge-feasibility checks, and resource adaptations remain proposed synthesis. ED, emergency department; SCAMP, Standardized Clinical Assessment and Management Plan; AHA, American Heart Association; ASE, American Society of Echocardiography; ACR, American College of Radiology.

## 3 Epidemiology and clinical context

Noncardiac diagnoses predominate, but estimates depend on case selection and definitions. In a study of two tertiary pediatric emergency departments, Drossner et al. identified a cardiac cause in 24 of 4,288 presentations after excluding children with known cardiovascular disease (7). A Portuguese emergency-department series reported cardiac etiologies in 1.1% of 798 visits, with musculoskeletal, idiopathic, and psychogenic diagnoses much more frequent (8). A 2024 meta-analysis reported a pooled cardiac proportion of 2.5%, but this combines heterogeneous cohorts and is not the probability of a life-threatening event in an individual child (9). A further emergency-department study of 10,721 encounters illustrates that testing patterns and the observed diagnostic mix can change over time, including during the COVID-19 pandemic (10).

Prehospital and cardiology populations should not be treated as interchangeable with unselected emergency presentations. The Victorian ambulance study included 4,277 attendances and found an association between abnormal initial physiology and serious short-term outcomes. Its diagnostic and outcome analyses used linked subsets of the cohort; the reported 1.6% serious-outcome frequency applies to the transported, linked hospital cohort, not all ambulance attendances. The outcome combined death, cardiac arrest, resuscitation-level triage, and intensive care admission rather than cardiac diagnoses alone (11). Referral cohorts likewise reflect prior clinical selection and different access to investigations (12, 13).

The practical inference is to assess both the clinical setting and the outcome being considered. A benign cardiac assessment does not exclude serious pulmonary or esophageal disease. Conversely, an incidental cardiac finding is not necessarily the cause of pain. Published percentages should therefore inform background expectations rather than function as universal risk thresholds.

## 4 Etiologic spectrum

### 4.1 Musculoskeletal and chest wall pain

Localized pain reproducible by palpation or movement often suggests a chest-wall source. Costochondritis, muscle strain, and precordial catch syndrome are common clinical considerations. A brief, focal episode at rest that worsens with inspiration may fit precordial catch syndrome, whereas swelling, significant trauma, systemic illness, or persistent focal bony pain requires a different assessment. Reproducibility alone does not override exertional syncope, abnormal physiology, or another concerning feature. When the complete presentation is reassuring, extensive investigations may offer little additional information, but imaging decisions should follow the clinical question and applicable guidance (1, 5, 12, 14).

### 4.2 Idiopathic and functional symptoms

An idiopathic diagnosis can be reasonable after an adequate assessment that finds no concerning explanation; it need not follow exhaustive exclusion by testing. Anxiety, panic symptoms, and other psychosocial difficulties may coexist with pain and functional impairment. These symptoms should be acknowledged without implying that the pain is unreal or that a psychological explanation excludes organic disease. Persistent symptoms warrant review of school participation, sleep, mood, and family concerns, with primary care or mental health support when appropriate (8, 15).

### 4.3 Respiratory and pleural disorders

Asthma, pneumonia, pleuritis, pneumothorax, and pneumomediastinum are important alternatives to cardiac disease. Acute pleuritic pain with dyspnea, hypoxemia, asymmetric breath sounds, or subcutaneous emphysema should prompt focused pulmonary assessment. Pulmonary embolism is uncommon but deserves consideration when compatible symptoms accompany relevant risks, such as thrombophilia, central venous access, malignancy, recent surgery or immobility, or estrogen exposure. Adult pulmonary-embolism rule-out strategies should not be assumed to have equivalent performance in children (5, 6, 14).

### 4.4 Gastrointestinal and systemic disorders

Reflux can cause meal-related or recumbent discomfort. Dysphagia, drooling, possible button-battery ingestion, or severe pain after forceful vomiting raises different and potentially urgent questions. Sickle cell disease with fever or respiratory symptoms requires assessment for acute chest syndrome, even when a cardiac cause seems unlikely. Known inflammatory disease, connective-tissue disease, or cardiotoxic exposure may also alter the differential and the threshold for specialist assessment (5, 6, 14).

### 4.5 Cardiac disorders

Myocarditis, pericarditis, arrhythmias, cardiomyopathy, and coronary anomalies are uncommon but clinically important. Exertional symptoms, syncope, sustained palpitations, an abnormal examination, or relevant personal and family history increase concern. Inflammation may present with persistent pain, systemic symptoms, disproportionate tachycardia, or electrocardiographic (ECG) changes; severe disease can present with heart failure or shock. A previous diagnosis of congenital heart disease, Kawasaki disease with coronary involvement, or inherited arrhythmia changes the interpretation of otherwise nonspecific symptoms (3, 7, 16). Table 1 summarizes clinical patterns and conditional assessment options; it is a differential-diagnosis aid rather than a diagnostic checklist.

## 5 Recognition of concerning features and provisional low risk

### 5.1 Physiological instability and stable warning features

Assessment begins with airway, breathing, circulation, mental status, and age-appropriate vital signs. Hypotension, poor perfusion, significant hypoxemia, severe respiratory distress, altered consciousness, or a clinically important sustained arrhythmia requires urgent stabilization and escalation. An initial apparently reassuring measurement does not remove the need to reassess a deteriorating child (5, 11).

In a stable patient, cardiac warning features include pain clearly precipitated by exertion, exertional syncope or presyncope, sustained palpitations, declining exercise tolerance, a pathological murmur, gallop or friction rub, and concerning personal or family history. Family history should clarify the circumstances and age of premature unexplained sudden death, cardiomyopathy, or inherited arrhythmia, particularly in first-degree relatives. Persistent tachycardia unexplained by distress or fever, abnormal pulses, and signs of heart failure also merit attention. These features guide further assessment; they do not independently diagnose cardiac disease or mandate the same test panel or disposition in every child (3, 4, 13).

### 5.2 What provisionally low risk means

In this framework, provisionally low risk means that the current assessment has not identified a plausible time-critical cause. It is a qualitative working assessment, not a numerical probability, a validated score, or proof that no disease is present. The following conditions should be considered together: an adequately obtained history; well appearance; reassuring age-appropriate vital signs, including repeat assessment when initially abnormal; a reassuring cardiopulmonary examination; no cardiac warning features; no acute pulmonary, esophageal, thromboembolic, or systemic concern; and a clinical pattern reasonably compatible with a benign explanation. These conditions synthesize established assessment principles rather than constitute a tested rule (3–5).

A lack of cardiac red flags alone is insufficient. The assessment is less secure when history is unreliable, a young child cannot describe symptoms, family history is unavailable, pain is severe or worsening without explanation, the examination is incomplete, or significant comorbidity changes baseline risk. Such uncertainty should prompt senior review, reassessment, targeted investigation, or referral according to the circumstances. A reassuring ECG, if obtained, cannot by itself exclude all coronary or structural abnormalities (3). A recent referral-clinic study reported a cardiac diagnosis in one of 316 children without its specified red flags, further illustrating that absence of those features is not synonymous with absence of disease; selective testing and diagnostic attribution limit interpretation (17).

Clinical risk and discharge feasibility should be assessed separately. A child may appear clinically low risk but still need observation or an alternative follow-up arrangement when reassessment cannot reliably be obtained. Conversely, lack of access to ECG, troponin, or echocardiography must not be treated as a normal test result. If an investigation is clinically indicated but unavailable, the question becomes how to obtain assessment or arrange transfer, not how to relabel the patient. This separation is a proposed implementation safeguard, not an independently validated predictor.

## 6 Diagnostic investigations and their limitations

### 6.1 Electrocardiography

ECG is useful when symptoms, examination, or history raise concern for a cardiac cause, and it can identify arrhythmia, conduction disease, repolarization changes, or clues to myocardial inflammation. It cannot reliably exclude all structural disease, coronary anomalies, or early myocarditis. The evidence supporting specialist chest-pain pathways often includes an ECG for all referred patients; that experience cannot automatically justify either universal ECG testing or universal ECG omission in primary care. In a clinically reassuring, clearly noncardiac presentation, deferring ECG may be reasonable under a local pathway, with reassessment if symptoms persist or evolve (3–5, 7).

### 6.2 Troponin and pretest probability

Troponin is a marker of myocardial injury, not a screening test for every cardiac cause of chest pain. Before requesting it, the clinician should specify the suspected process and how the result could change management. A plausible concern for myocarditis or myopericarditis, myocardial ischemia, or injury associated with a significant arrhythmia is more relevant than chest pain alone. Supporting features may include persistent pain with systemic illness, unexplained tachycardia, dyspnea, poor perfusion, or compatible ECG abnormalities. These are contextual indicators, not a validated combination or a requirement that all be present (16, 18).

Selection strongly affects apparent test yield. Brown et al. studied 212 chest-pain patients younger than 22 years who had already been selected for troponin testing: 37 had elevated results, and 18 of those had a primary cardiac diagnosis. These proportions are not estimates of performance in all children presenting with chest pain (19). A multicenter study of pediatric troponin use similarly found that interpretation depended on the presentation and accompanying findings (20). At a lower pretest probability of myocardial inflammation, a positive result has a lower positive predictive value for that diagnosis, even if sensitivity and specificity are unchanged. An isolated mild elevation may therefore trigger additional testing without clarifying the cause of pain. Some elevations represent genuine myocardial injury from another illness rather than an analytically false result (2). Assay interference is a separate possibility when results and the clinical picture are discordant; laboratory consultation can help resolve such discrepancies (21).

In a higher-probability presentation, an elevated troponin supports myocardial injury but does not establish its etiology. A normal initial result does not by itself exclude early or limited myocarditis, pericarditis without myocardial involvement, or structural cardiac disease. Persistent concern warrants clinical reassessment and consideration of repeat testing, formal echocardiography, or specialist assessment. Repeat sampling should follow symptom timing, evolution, assay characteristics, and local pediatric advice; adult rapid acute-coronary-syndrome algorithms and fixed serial-testing schedules should not be imported uncritically (3, 16).

Assay-specific units and reference limits must be checked. Pediatric reference studies show that upper limits vary by assay and reference population, with age and sex relevant to interpretation (22). A 2026 multicenter cohort showed that applying a pediatric 97.5th-percentile threshold classified more tested patients as having myocardial injury, but the clinical benefit of this reclassification remained uncertain (23). We therefore do not propose a universal troponin cutoff, delta, or discharge threshold.

The myocarditis prediction study by Genadry et al. also requires careful interpretation. It retrospectively compared 93 cases with 202 controls among patients already tested for suspected myocarditis. At least one of chest pain, fever, or specified ECG changes yielded 99% sensitivity but 14% specificity in that selected sample. Because chest pain is itself a predictor, the one-predictor threshold cannot identify a low-risk subgroup within a population defined by chest pain. Case-control sampling also prevents direct estimation of prevalence and predictive values in an unselected population. The study should not be used to justify omitting troponin or further assessment in all chest-pain patients; external prospective evaluation is needed (24).

### 6.3 Chest radiography

Chest radiography can address suspected pneumothorax, pneumomediastinum, pneumonia, acute chest syndrome, or other thoracic disease. In a two-center French series of 599 children with nontraumatic chest pain, clinical features were associated with abnormal radiographs, but the small number of abnormal studies limited prediction modeling (25). This supports attention to clinical indications, not a universally applicable radiography rule.

Guidance also differs in scope. The Royal Children's Hospital pathway reserves investigations for risk factors identified on history and examination, whereas the ACR chest-pain criteria rate radiography as usually appropriate for initial imaging of pain localized to the chest wall and for suspected pneumothorax or pneumomediastinum (5, 6). These documents answer partly different questions. Accordingly, we suggest selecting imaging for a defined clinical concern and the applicable local guidance, rather than stating that radiography is never indicated in a reassuring presentation. A chest-wall pain label should not obscure persistent focal symptoms, trauma, systemic illness, or diagnostic uncertainty.

### 6.4 Echocardiography and advanced cardiac imaging

Formal echocardiography is useful when examination, ECG, symptoms, or background disease raises concern for structural disease, ventricular dysfunction, pulmonary hypertension, or pericardial effusion. It generally has limited additional yield when the entire presentation is reassuring, but referral pathways differ in their thresholds. Coronary imaging or cardiac magnetic resonance should address a specialist-defined question. Neither a normal ECG nor a limited bedside ultrasound can replace a formal study when the suspected diagnosis requires it (3, 4, 6).

### 6.5 Ambulatory monitoring and exercise testing

Monitoring is most informative when there is a plausible intermittent rhythm disturbance, particularly palpitations or syncope. Test yield must be interpreted according to its definition: a recent Holter study counted capture of symptoms without arrhythmia as a diagnostic result, so its overall yield is not the proportion with pathological arrhythmia (26). Routine monitoring or exercise testing for isolated reassuring chest pain is generally difficult to justify, whereas selected exertional or rhythm-related presentations may warrant specialist-directed testing. A negative exercise test does not exclude every coronary anomaly (3, 4).

### 6.6 POCUS as an optional adjunct

POCUS may help a trained clinician answer a focused question about ventricular systolic function, pericardial fluid, or a pulmonary abnormality. Professional recommendations emphasize pediatric-specific training, image quality, documentation, and quality assurance (27). Direct chest-pain evidence remains limited. A prospective pneumothorax pilot included 77 selected children and 13 pneumothoraces; enrollment depended on experienced operators being available, and chest radiography was the reference standard. Its encouraging accuracy estimates should not be generalized to unselected chest pain or interpreted as a validated discharge strategy (28). Cardiac POCUS data in children with preexisting cardiac disease address a different population and used expert review of POCUS images as the comparator (29).

POCUS is therefore optional within the proposed framework, not a routine screening step or a replacement for indicated radiography or formal echocardiography. A negative or technically inadequate scan cannot exclude coronary anomalies, all structural disease, or myocarditis. Persistent concern requires further assessment regardless of the bedside scan. Availability of a machine without trained staff is not adequate diagnostic capability, and scanning must not delay resuscitation or transfer. The ACR imaging guidance for suspected pneumothorax or pneumomediastinum favors radiography rather than routine initial chest ultrasound, reinforcing the need for local, indication-specific use (6, 27).

## 7 Evidence from standardized clinical pathways

The strongest implementation experience concerns defined pathways in particular health systems, not the framework proposed here. The initial Standardized Clinical Assessment and Management Plan (SCAMP) study retrospectively applied an assessment algorithm to 406 cardiology referrals and estimated potential reductions in selected tests (4). In a subsequent comparison of 364 SCAMP patients with 406 historical controls, exercise testing and rhythm monitoring decreased; overall echocardiography use did not significantly change (30). A later assessment found no cardiac pathology judged to have been missed at the initial SCAMP encounter, but retrospective ascertainment and rare events limit the certainty of that safety finding (31).

Emergency and primary care evaluations also suggest context-specific benefits. In a pediatric emergency-department study of 1,687 eligible patients, pathway implementation reduced chest-radiograph ordering; other testing and cardiology consultation did not change materially. Follow-up within that health system was incomplete (32). A quality-improvement initiative in four primary care practices reduced low-probability referrals from 17% to 5%, with no life-threatening cardiac event identified over a median 0.9 years of follow-up (33). Such findings support structured assessment and local audit, but do not demonstrate zero residual risk or an identical benefit in other settings. No universal percentage reduction in investigations or costs is attributed to our proposed framework.

## 8 Proposed evidence-informed clinical framework

Figure 1 organizes assessment into four management categories. These are not numerical risk strata, mutually exclusive diagnoses, or a validated clinical decision rule. No patient cohort was used to derive predictors or thresholds for this framework; its sensitivity, specificity, calibration, and clinical impact have not been measured. Evidence supporting individual components or existing SCAMP pathways does not validate their combination here. A child may have both cardiac and noncardiac concerns, and unexplained or evolving symptoms require reassessment rather than forced assignment to the low-risk category.

**Figure 1:**
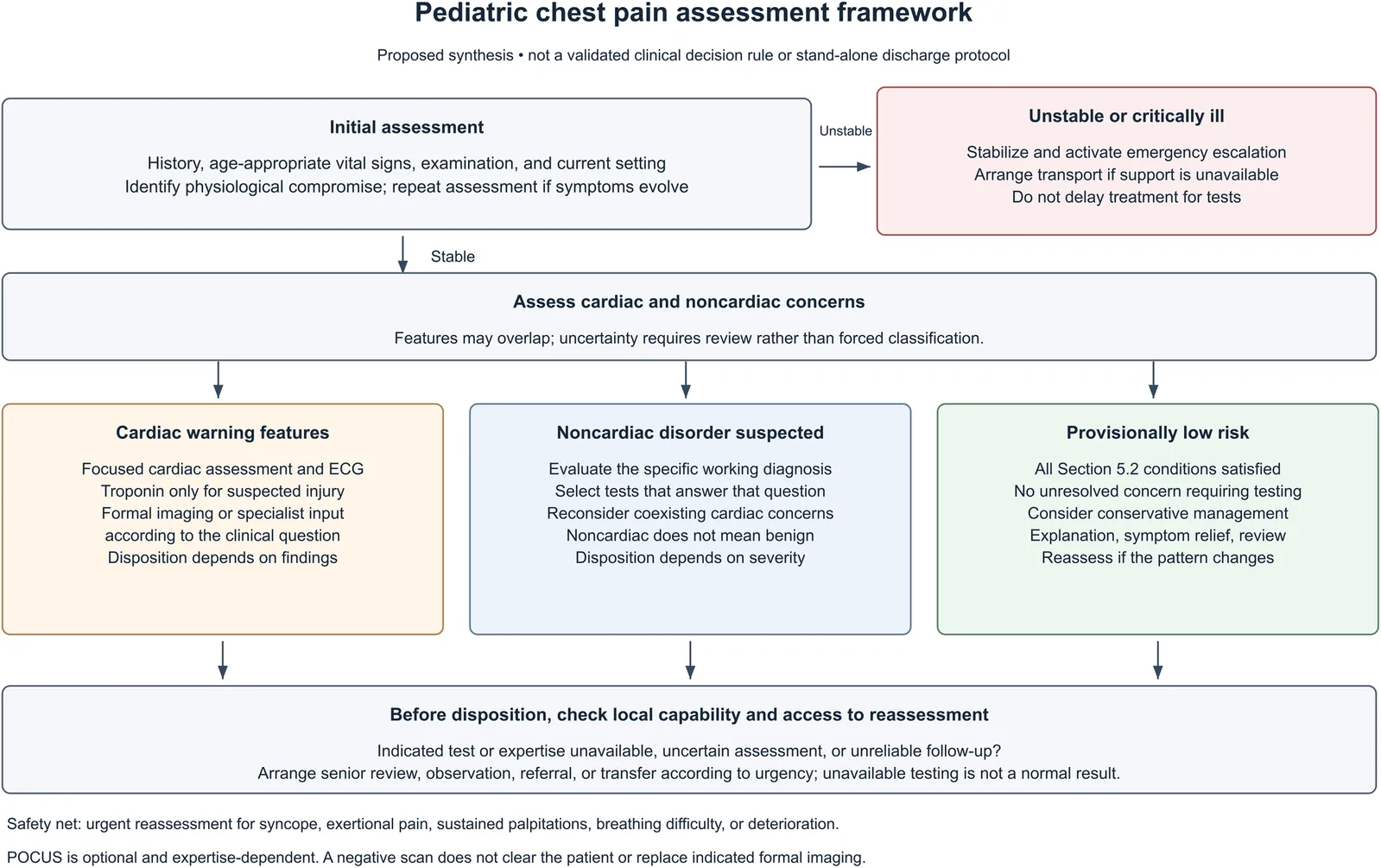
Proposed evidence-informed framework for pediatric chest pain assessment. The framework has not been prospectively derived or externally validated. Its categories guide clinical reasoning and are not mutually exclusive diagnoses or quantitative risk strata. Apply the complete provisional low-risk definition in Section 5.2; an inadequate assessment or unresolved concern requires further review. Clinical setting, available expertise, and access to reassessment influence disposition but do not change biological risk. This is not a prehospital nontransport rule. POCUS is an optional adjunct and must not delay definitive care. ECG, electrocardiography; POCUS, point-of-care ultrasound.

### 8.1 Physiologically unstable or critically ill

Begin condition-directed stabilization, monitoring, and emergency escalation according to pediatric resuscitation protocols. Tests should be obtained when they can inform immediate care without delaying treatment. Where definitive support is unavailable, activate emergency transport or retrieval early. This category concerns urgency rather than a presumed cardiac diagnosis (5, 11).

### 8.2 Stable with cardiac warning features

Obtain or arrange timely ECG and focused cardiac assessment according to the specific concern. Troponin is conditional on suspected myocardial injury, rather than automatic for every warning feature. Formal echocardiography, rhythm assessment, and specialist advice should be selected accordingly. Admission or monitored observation may be warranted for significant abnormalities, ongoing concerning symptoms, or an evolving illness. Some otherwise stable patients may be suitable for expedited outpatient assessment when the clinician is satisfied that immediate care is unnecessary and follow-up is reliable. Exertional symptoms or suspected cardiac disease may justify avoiding strenuous activity until assessment (3, 4, 16).

### 8.3 Stable with features of a noncardiac disorder

Direct evaluation toward the suspected pulmonary, chest-wall, gastrointestinal, or systemic condition, while reconsidering coexisting cardiac concerns. Radiography or other investigations should answer a defined question; POCUS may supplement assessment where expertise and local protocols support it. Testing, treatment, and disposition depend on the suspected disease and severity rather than on the label noncardiac. Suspected embolism, esophageal obstruction or perforation, and significant respiratory disease may require urgent specialty care (6, 14).

### 8.4 Provisionally low risk

When the conditions in Section 5.2 are met and no unresolved clinical question requires investigation, it may be reasonable to avoid routine broad laboratory panels, troponin, echocardiography, or rhythm monitoring. ECG and radiography remain conditional on the clinical question and local guidance. Explain the working diagnosis, offer appropriate symptom relief, and discuss activity according to symptoms and any unresolved concern. Provide explicit instructions to seek urgent reassessment for syncope, new exertional pain, sustained palpitations, breathing difficulty, deterioration, or persistent or worsening pain. Arrange review for unresolved symptoms. If the family cannot access reassessment or understand the plan, address that barrier before deciding on discharge (3–5, 32).

## 9 Clinical setting and resource availability

The same symptom can require different next steps in different services. In primary care, longitudinal history and access to follow-up may support conservative management after an adequate assessment, while time-critical features require emergency referral. Emergency departments can observe symptom evolution and arrange targeted tests, but pediatric ECG interpretation and specialist access vary. Cardiology clinics see a referral-selected population; their use of routine ECG cannot be assumed to apply unchanged to every community presentation (4, 32, 33). Prehospital care should prioritize physiology, stabilization, destination, and safe transport under local protocols. This review does not validate an ambulance nontransport rule (11).

Table 3 distinguishes setting from available resources and presents proposed implementation considerations. A basic assessment requires a reliable history where possible, vital signs, examination, recognition of deterioration, and a means of obtaining further help. If an indicated ECG, biomarker assay, imaging study, or interpretation service is unavailable, telephone or telemedicine advice may help determine where and how urgently assessment should occur, but it cannot replace necessary in-person investigation. Suspected myocarditis with worsening symptoms should not wait for an unavailable local troponin assay; similarly, bedside POCUS should not be treated as a substitute for formal coronary assessment.

**Table 3:** Proposed adaptations to clinical setting and available resources.

Setting or constraint	Main assessment task	Practical approach	Safety boundary
Primary care or community clinic	Separate a reassuring presentation from a time-critical concern; use longitudinal history when available.	Assess history, vital signs, examination, and follow-up access. Arrange ECG or specialist assessment when indicated; provide a specific review plan for persistent symptoms.	Instability or suspected acute serious disease warrants emergency referral, not a routine outpatient appointment.
Emergency department	Reassess physiology and symptom evolution; address targeted diagnostic questions.	Select ECG, laboratory studies, and imaging according to the working diagnosis. Use observation and senior review when uncertainty remains.	A reassuring test cannot override a concerning history or clinical deterioration. Pediatric interpretation expertise may be required.
Pediatric cardiology clinic	Assess a referral-selected population for cardiac causes and activity-related concerns.	Interpret prior testing and decide whether formal echo, rhythm recording, or other specialist investigations will change care.	A referral-clinic pathway with routine ECG cannot be assumed to validate omission of ECG in another setting.
Prehospital service	Recognize compromise, stabilize within scope, and select destination and transport urgency.	Repeat vital signs; obtain ECG only where available and appropriate without delaying transport. Follow local pediatric transport protocols.	The proposed low-risk category is not validated for a decision to leave a child at scene.
Limited tests, specialist access, or follow-up in any setting	Identify what information is needed and whether it can be obtained safely and promptly.	Use telephone or telemedicine advice where feasible; arrange assessment at an appropriate facility when an indicated test is unavailable. Consider observation or earlier transfer when reassessment is unreliable.	Unavailable testing is not normal testing. Lack of trained POCUS staff must not block care; a bedside scan is not a replacement for required formal imaging.

These are proposed implementation considerations, not validated setting-specific rules. Relevant supporting context comes from primary care, ED, cardiology, prehospital, and professional guidance sources (4, 5, 11, 27, 32, 33). Local resources affect the feasible care plan, not the patient's biological risk. No universal admission, transfer, or follow-up time threshold is established by this review.

Resource constraints affect the feasibility of management, not the child's biological risk. A well-resourced center should not test without a clinical question merely because a test is available. A resource-limited service should not classify unresolved concern as benign merely because tests are unavailable. Long travel distances, limited monitoring, uncertain follow-up, and barriers to emergency return may justify longer observation, earlier consultation, or transfer. These are pragmatic safeguards proposed for local evaluation, not interventions with a demonstrated effect size in pediatric chest-pain trials.

## 10 Limitations and priorities for evaluation

This review is a selective narrative synthesis with the search limitations described in Section 2. Many influential studies are retrospective, referral-based, or restricted to patients already selected for testing. Inconsistent definitions of cardiac causation, incomplete verification of untested patients, and variable follow-up can bias prevalence and diagnostic-yield estimates. Small numbers of serious events limit claims about safety even in larger cohorts. Guidance may differ because it addresses different clinical questions or relies partly on expert opinion. The limited direct evidence for POCUS and the uncertain implications of pediatric troponin thresholds are particularly important examples.

The proposed framework adds an explicit account of applicability and uncertainty, but its value remains to be established. Prospective evaluation should first assess feasibility and interclinician agreement on the categories and low-risk conditions. Subsequent multicenter work should use predefined serious cardiac and noncardiac outcomes, blinded outcome adjudication where feasible, follow-up sufficient to detect delayed diagnoses, and ascertainment beyond a single hospital network. Testing rates, avoidable referrals, return visits, family understanding, and inequities in access should accompany safety outcomes. Community, prehospital, and resource-limited settings require direct evaluation rather than extrapolation from tertiary cardiology. If the framework is converted into a formal decision rule, separate derivation and external validation are necessary before claims of rule-out performance are made.

## 11 Conclusion

Pediatric chest pain is commonly benign, but selective evaluation requires more than the absence of an obvious cardiac red flag. Clinical context, an adequate assessment, the pretest probability of the condition being investigated, and access to reassessment all matter. Troponin and imaging should answer specific questions, and POCUS remains an optional adjunct. The proposed framework connects these considerations and makes their evidentiary limits explicit. It is a narrative synthesis to support clinical reasoning and future evaluation, not an evidence-validated decision rule or a stand-alone discharge protocol.
